# Is legal status associated with mental illness among newly arrived refugees in Sweden: an epidemiological study

**DOI:** 10.1186/s12888-023-04679-y

**Published:** 2023-03-24

**Authors:** Sara Delilovic, Ana Hagström, Jad Shedrawy, Anna Clara Hollander, Knut Lönnroth, Henna Hasson

**Affiliations:** 1 Procome Research Group, Department of Learning, Informatics, Management and Ethics, (LIME) Karolinska Institutet (KI), Karolinska Institutet, Region Stockholm, 171 77 Sweden; 2 Region Stockholm, Centre for Epidemiology and Community Medicine (CES, with Swedish acronym), Region Stockholm, Sweden; 3 Social medicine, infectious diseases and migration, Department of Global Public Health, Karolinska Institutet, Solna, Sweden; 4 Epidemiology of Psychiatric Conditions, Substance use and Social Environment (EPiCSS), Department of Global Public Health, Karolinska Institutet, Solna, Sweden

**Keywords:** Refugees, Asylum seekers, Mental health, Screening, Primary care

## Abstract

**Background:**

There are about 80 million forcibly displaced people globally. Migrants are at heightened risk for mental illness compared to host country populations. While previous research highlights the need to adequately assess mental illness, few have taken the diversity among newly arrived migrants into account. This study aims to estimate the prevalence and associated risk factors of mental illness among asylum seekers, quota and other refugees in Stockholm, Sweden.

**Methods:**

Using a cross-sectional design, data was collected as part of a mental health screening initiative integrated into routine health examinations in two health care clinics in Stockholm. Screening was done with the Refugee Health Screener, RHS-13, a validated instrument for assessing mental health in refugee populations.

**Results:**

A total of 1163 individuals were eligible for screening, of whom 566 participated (response rate 48.6%). Among the participants, 47.9% indicated symptoms of mental illness. Compared with asylum seekers, the risk of mental illness was lower among quota and other refugees (adjusted odds ratio 0.60, 95% confidence interval 0.37-1.00). Female sex, higher age, coming from a middle-income country and low probability of being granted asylum were significant predictors of mental illness.

**Conclusion:**

Refugee legal status is associated with mental illness. Asylum seekers are at greater risk of mental illness compared to quota and other refugees. Our findings call for screening for mental illness among newly arrived migrants, especially among those with pending residence permits.

## Background

In recent years, wars, conflicts and violation of human rights have led to forced displacements, with millions of people seeking protection outside of their home countries [1]. The global population of forcibly displaced people grew substantially from 43.3 million in 2009 to 70.8 million in 2018 [1]. In 2017–2018, Sweden hosted the largest number of refugees in the European Union (EU) per capita [2–4] and today, in 2022, there are millions of Ukrainian people fleeing their homes seeking safety and protection.

From a legal perspective, an asylum seeker is a person who seeks sanctuary and files for refugee status in a country other than their own and awaits a decision, while a refugee is someone who has been recognized under the *1951 Convention relating to the status of refugees* to be a refugee [5]. In the later definition, quota refugees are included as those who have been selected by the UN´s refugee agency, United Nations High Commissioner for Refugees (UNHCR), to be resettled to a third country that offers them protection. Resettling in a third country is a solution for people who can neither remain in their first country of asylum nor return home. Unlike asylum seekers, quota refugees have a residence permit upon arrival. The same applies for other non-refugee migrants, such as those coming for family reunification [3, 5].

Migrant-specific social determinants of health are often separated into pre-, peri- and post-migration factors [6, 7]. Pre-migration factors refer to factors before migration such as social disadvantage in the country of origin or conflict-related traumatic experiences. Peri denotes the stage with factors related to the physical transition and journey.

Post-migration factors include a poor social network in host country, acculturation and adaptation barriers, and overlap with general social determinants of health [6, 7].

The estimates of the prevalence of mental illness in refugees varies. A recent systematic review of peer-reviewed articles of the mental health among Syrian refugees resettled in 10 countries found a prevalence rates of 43% for PTSD, 40% for depression, and 26% for anxiety [2, 8]. Furthermore, a systematic review and meta-analysis including refugees from 15 countries, found rates of 31.46% for PTSD, 31.5% for depression and 11% for anxiety disorders [9]. In Germany, one of the countries hosting the largest number of refugees in Europe, a meta-analysis found differences in prevalence depending on type of screening method with regards to depression but not to PTSD, with significantly higher prevalence estimates for depressive symptoms reported using screening tools (39.8%) in comparison with diagnostic instruments (28.4%), whereas survey methods provided comparable prevalence estimates for symptoms of PTSD (28.1% v. 29.9%) [10]. In addition to screening methods used, the variations in prevalence have also been found to be impacted by length of stay in host country, timing of screening and legal status of the person [10]. Legal status in this context refers to the legal recognition of the person, ex: asylum seeker, refugee, stateless person.

So far, many of the diagnostic measures have been widely used in different cultural contexts, but none have still been specifically developed for refugee populations or cross-cultural use [9, 11].

Unlike quota refugees, asylum seekers have not experienced an organized resettlement and studies have found that the asylum process, particularly long asylum processes, discrimination and post-migration stress in the host country are associated with an increased risk of mental illness. This includes lack of social networks, less favorable living conditions, lack of working opportunities and access to health care in host country. Along with the above-mentioned factors, sex and education attainment have shown to be associated with the risk of mental ill health among refugees [11–13].

With regards to quota refugees, evidence suggests that even though quota refugees experience organized settlement, they have often been forced to live in refugee camps for long periods before being selected for resettlement. While refugee camps can be a safe escape, quota refugees are likely to have experienced disadvantaged living conditions for long periods. Refugee camps are often dangerous and may have higher mortality rates than countries of origin due to interethnic conflict, sexual violence, disease epidemics and lack of health care access, which impose a higher risk for mental illness [12, 14, 15]. Studies have also found age, sex, and region of origin to be relevant factors for the groups of quota refugees in terms of mental illness outcomes. [12, 13, 15].

A recent longitudinal study among Syrian quota refugees in Norway found mental illness improvements in both anxiety/depression and post-traumatic stress disorder over time. The authors found short length of stay in host country, as well as younger age modified the improvements [15]. The variation in prevalence of mental illness among refugees could be explained by differences in screening methods applied but also differences in the characteristics of the populations. Additional reasons for wide variability could be the lack of differentiation between different groups of newly arrived people such as asylum seekers and refugees, including both quota and other refugees.

There is no routine protocol for addressing mental illness in asylum seekers and other refuges upon arrival in Sweden. In this study, we screen asylum seekers and other refugees with the Refugee Health Screener (RHS-13) at a free-of-charge health examination in primary health clinics as a first step to overcome the issue. The approach of choosing screening over other assessment methods, such as diagnostic testing, is that it a) has a low threshold, (b) is easy to implement in the existing structure (i.e., free-of-charge health examination in primary health clinics) and (c) it is likely to be cost-effective. In addition, screening with the RHS-13 allows for assessment of symptoms of the most common mental health problems in refugees: PTSD, depression, and anxiety, rather than presence of a specific mental health condition.

The aim of this study is to estimate the differences in prevalence of mental illness between asylum seekers and refugees, including both quota and other refugees and to determine if sex, age, gross national income per capita in the country of origin, or country of origin (based on the asylum recognition rate) could explain these differences.

We hypothesize that the prevalence of mental illness differs between asylum seekers and quota refugees, assuming that the prevalence is higher for those with pending legal status and women. We also hypothesize that the differences could partly be explained by age, gross national income per capita in the country of origin and country of origin (based on the asylum recognition rate). Our specific research questions are:

- What is the prevalence of mental illness, measured by RHS-13 among asylum seekers and newly arrived refugees?
- Are there differences in the prevalence and likelihood of mental illness in respect of migration status, sex, age, gross national income per capita in the country of origin and country of origin (by asylum recognition rate).
- To what extent does legal status impact mental illness measured with RHS-13.

## Method

### Setting

The study was conducted in two primary health care clinics in Stockholm Region, Sweden.

In Sweden, all asylum seekers and refugees are offered a free-of-charge health examination (HE) in primary health care clinics. The HE aims to identify health care needs, including mental and physical health problems that require immediate attention and to detect infectious diseases that require special control measures [16, 17]. The patient coming for HE does not need to be symptomatic or have symptoms for any condition/disease.

The HE if offered as a way to get in contact with the health care system. The organization around the HE involves different entities. The content and recommendations for what a HE should include (minimum requirements) are regulated by the National Board of Welfare and the Public Health Agency [18]. Training is provided to health care professionals conducting the HE. The HE is voluntary and in terms of content, the patient chooses what health related information he or she wants to share during the HE. The HE is always performed in the patient preferred language, with or without an interpretation if the health care professionals are not native speakers.

Until now, healthcare professionals in the Stockholm region have not been using standardized methods to address mental health during the HE [16], compared to other parts of the HE such as screening for infectious diseases and vaccinations, where recommendation/guidelines are in place.

### Design and population

We introduced a validated screening instrument, the RHS-13, discussed further under the sub-section “Outcome” [19].

The RHS-13 screening instrument was implemented and integrated within the HE. Screening was performed by the healthcare professional conducting the HE during a period of 6 months and registered in the electronic medical record system (October 2018- March 2019). Equal training was given to all health professionals, with a one-day workshop on how to administrate the RHS-13 in the HE, to make the procedure as standardized as possible.

All screening took place either in their native language, if the RHS-13 was available, or with interpretations. Using a cross-sectional design, data for this period was extracted during September 2019. The two clinics accounted together for more than half of all HE conducted in the region during this period. All migrants, age 14 or older were eligible for the screening. Not all participants completed screening with the RHS-13. Out of all individuals who came for a HE during the study period, 597 (51,4%) were not screened. Reasons for no screening were not recorded.

Six individuals were excluded from the analysis due to not meeting the inclusion criteria of being 14 years of age or older and 9 were excluded due to missing data on RHS-13.

### Ethical issues

Ethical clearance was obtained from the Swedish Ethical Review Authority (Dnr: 2019–01408) to retrieve HE data from medical records (MR). All data was recoded and pseudonymized, meaning data items were recorded to an artificial identifier (a pseudonym). This step was done on site at the health care clinic to ensure privacy. Data were coded into excel files.

#### Informed consent

was obtained from participants once they came to the HE, otherwise they would opt out/decline to participate. For those under the age of 15, informed consent was obtained from a parent and/or legal guardian. Data that was recorded within the HE is handled according to Swedish law, in the same way as for all citizens. Information on how data is handled is given in the information letter given by the region and orally once the patient comes to a HE. The HE is voluntary and in terms of content, the patient chooses what health-related information he or she wants to share during the HE, meaning they can opt out. Consent to use the data from the HE was secured by the Swedish Ethical Review Authority. The data is equivalent to data from databases/registries and data was only handled and accessible to authorized persons.

### Variables

#### Outcome

The outcome was prevalence of mental illnesses which was measured with RHS-13. RHS-13 is a screening tool to identify individuals with symptoms of mental illness (i.e., not a diagnostic tool) [19–21]. RHS-13 consists of 13 questions and assesses symptoms of the most common mental health problems in refugees: PTSD, depression, and anxiety. The original version, consisting of 15 items, shows excellent internal consistency (Cronbach´s a = 0.91) (PTSD: sensitivity 0.81/specificity 0.87, anxiety 0.94/0.86, and depression 0.95/0.89) [19]. A 13-item version of the RHS-15 has been tested using diagnostic proxy instruments, which showed strengthened psychometric properties (Cronbach´s a = 0.96) without comprising the validity of the instrument [20, 21]. Considering the Swedish context, the 13-item version was assessed in 2017 among asylum seekers coming from Afghanistan, Syria Iraq, Iran, Somalia, and Eritrea, showing good internal consistency (Cronbach´s a = 0.92) for the total sample. We did not test for internal consistency in our study, as it was tested in the Swedish context on a similar sample (in terms of country of origin and other demographic variables) and close in time to when our data was retrieved.

RHS-13 has been validated and is available in 18 languages including Swedish [21].

The RHS-13 scale is answered with a five-point Likert scale, with scores from 0 to 4, corresponding to “Not at all” to “Extremely”. The total range of scores is thus 0–52 [19, 20]. A cut-off of ≥ 11 has been suggested to identify positive screening for mental illness. The instrument has also been tested in Sweden for severity levels of symptoms of psychological distress where a score of 11–17 is interpreted as mild symptoms, 18–24 as clinically significant problems (moderate), and 25 or above as acute and severe problems [19, 21]. We present prevalence of mental illness based both on dichotomized scores (positive 11–52 vs. negative 0–10), as well as based on cut-offs for mild (11–17), moderate (18–24) and severe (25–52) problems.

#### Exposure

Our primary exposure was legal status, categorized as “Asylum seekers” or “Quota and Other refugees”.

The group called “Others” accounts for individuals who have entered Sweden for family reunification knowing that they would qualify for a residence permit as they have one or more family members who have been granted permit as a refugee or a person in need of subsidiary protection (i.e., someone who does not qualify as refugee but would face a real risk of suffering serious harm in his or her home country). Individuals with family reunification were merged into the group “Quota and Others” as we expected them to share similar characteristics and legal benefits as quota refugees.

#### Predictors

Sociodemographic measures.

Sex was categorized into men and women.

Age was categorized into the following groups: 14–25 years, 26–35 years and 36 and older.

Gross national income per capita (GNI) is the dollar value of a country’s income in a year, divided by its population. GNI presents a country’s economic strengths and is closely linked to indicators on an individual level such as social, economic and environmental well-being. Countries with higher GNI, tends to have higher literacy rates and longer life expectancies. Country of origin by World Bank GNI Classification was defined as:

- low-income country ($1.025 or less). Example of countries in our study: Afghanistan, Ethiopia, Eritrea, Somalia, and Syria.
- lower-middle income country ($1,026-$3.995). Example of countries in our study: Mongolia, Kenya, Uzbekistan, and Morocco.
- upper-middle and high ($3.995 or more) income country. Example of countries in our study: Iran, Iraq, Georgia, Turkey, and Algeria.

Data on GNI classification was retrieved from the World Bank and reflects classifications made in 2020 [22].

Country of origin by probability rate of first-time recognition reflects the recognition rate, the share of positive decisions among the total number of decisions by citizenships of asylum applicant in the EU. Rates account for positive decisions from the 4th quartile in 2017 to the 4th quartile in 2018 for refugee status, subsidiary protection and humanitarian reasons in the EU [23].

- Low probability rate was defined as 0–39%.
- Middle 40–79%.
- High 80–100%.

### Statistical analyses

Sociodemographic variables were described by absolute and relative frequencies. Chi^2^-test were used for associations between categorical independent variables. Prevalence (%) of positive screenings for mental health symptoms by sociodemographic characteristics were estimated with 95% Confidence intervals (CI). Regression models were performed on the dichotomized outcome, defined as RHS-13 ≥ 11, as it is more established and used than the ordinal outcome (with 3 severity levels). Logistic regression was used to control the association between the outcome and exposure for potential confounders. Stepwise modelling, using a bidirectional elimination method was completed [24]. Backward elimination and forward selection approaches were conducted. We tested all independent variables incrementally for significance. All significant variables were imputed in the final model and variables were removed to test for significance. We tested for interaction between all independent variables. One significant interaction was found, between sex and GNI classification. Stratified analysis by sex is presented for both crude and adjusted models. Two adjusted models are presented. Model 1 was adjusted for age, sex, and probability of getting asylum, leaving GNI classification out, considering it to be a priori confounder. Model 2 was adjusted for age, sex, probability of getting asylum, GNI classification as well as the interaction between sex and GNI classification. Interaction variables were not used in stratified models.

Results are presented for crude and adjusted odds ratios (ORs) with 95% confidence intervals. A p-value of 0.05 was considered statistically significant. Statistical analyses were performed using IBM SPSS version 26 [25]. For the purpose of the study, we have selected asylum seekers as the reference group, as they account for the largest number of observations in our sample.

## Results

Out of the 1163 respondents who came and underwent a health examination, 566 completed screening with the RHS-13 (48.6%). The majority were men and the largest group by country of origin were Syrians followed by Eritreans, Iranians, and Uzbeks. In total, 78 countries were represented. Majority of the cohort came from a low-income country and with a low probability rate of first-time recognition (Table 1).

**Table 1:** Sample Characteristics of respondents

Sample Characteristics		
Total	**N = 566**	
	n	(%)
**Clinic**		
Clinic 1	246	49.7
Clinic 2	320	47.9
**Sex**		
Men	354	62.5
Women	212	37.5
**Legal status**		
Asylum seekers	393	69.4
Quota and other refugees	171	30.2
**Age**		
14–25	183	32.3
26–35	170	30.0
36>	213	37.6
**Country of origin by World Bank GNI Classification**		
Low	227	40.1
Lower-middle	152	26.9
Upper- middle and high	186	32.9
**Probability rate of first-time recognition**		
Low (0–39%)	319	56.4
Middle (40–79%)	118	20.8
High (80–100%)	129	22.8
**Country of origin**		
Syria	78	13.8
Eritrea	51	9.0
Iran	51	9.0
Uzbekistan	30	5.3
All Others	356	62.9

In total, 47.9% scored positive for symptoms of mental illness (score ≥ 11) (Table 2). A score ≥ 11 was significantly more common among asylum seekers than quota and other refugees (χ^2^ = 14.21 df= (1), p = 0.00) (Table 2). Women (χ^2^ = 13.96 df= (1), p = 0.00), older age groups (χ^2^ = 7.55 df= (2), p = 0.02) and people from a middle- or high-income country (χ^2^ = 11.95 df = (2), p = 0.00) had significantly higher prevalence of a score ≥ 11. The prevalence of score ≥ 11 was similar for individuals with a low and middle probability of getting a positive decision on their asylum application, but significantly lower for those with a high probability (χ^2^ = 14.17 df = (2), p = 0.00). Significant differences were found in the prevalence of severe mental illness (score ≥ 25) in the univariate comparisons regarding sex (χ^2^ = 19.34 df= (3), p = 0.00), age (χ^2^ = 22.42 df = (6), p = 0.00), country of origin by GNI classification (χ^2^ = 18.07 df= (6), p = 0.00) and probability rate of first-time recognition (χ^2^ = 23.39 df= (6), p = 0.00 (Table 2). The prevalence of severe symptoms was about twice as high for women compared to men, asylum seekers compared to quota and other refugees, older population compared to younger population, and those from middle-income compared to low-income countries. Those from countries with a low probability of first-time recognition had about three times higher prevalence of severe problems than those with high probability.

**Table 2:** Prevalence (%) of mental illness measured with RHS-13 in total and by sociodemographic characters, country of origin by World Bank GNI classification and by probability rate of being granted protection status in EU.

Mental Illness measured with RHS-13					
	Mild mental illness (≥ 11–17)	Moderate mental illness (≥ 18–25)	Severe mental illness (≥ 25)	Any mental illness (all ≥ 11)	No mental illness (all < 11)
	**n = 74**	**n = 73**	**n = 124**	**n = 271**	**n = 295**
	%	%	%	%	%
**Total**	13.1	12.9	21.9	47.9	52.1
Sex*					
Men	13.3	11.9	16.7	41.8	58.2
Women	12.7	14.6	30.7	58.0	42.0
Legal Status*					
Asylum seeker	13.7	13.2	26.0	52.9	47.1
Quota and other refugees	11.7	11.7	12.3	35.7	64.3
Age **					
14–25	13.1	11.5	15.3	39.9	60.1
26–35	15.3	17.1	17.1	49.4	50.6
36>	11.3	10.8	31.5	53.5	46.5
Country of origin by World bank GNI Classification*					
Low	13.0	12.2	13.9	39.1	60.9
Lower-middle Upper-middle and high	12.7 13.4	15.3 11.8	26.7 28.0	54.7	45.3
				53.2	46.8
Probability rate of first-time recognition*					
Low (0–39%)	13.8	14.1	24.1	52.0	48.0
Middle (40–79%)	11.0	11.0	30.5	52.5	47.5
High (80–100%)	13.2	11.6	8.5	33.3	66.7

*p value < 0.00, **p-value < 0.05

Stratified by sex, asylum seeking men and women still had double the likelihood of mental illness as compared with quota and other refugee men (OR = 0.55 CI 0.33–0.89, p = < 0.01) and women (OR = 0.36 CI 0.20–0.6, p = < 0.00) (Table 3). Stratified by legal status, asylum seeking men still had about half the likelihood of mental illness compared to women (OR = 0.43 CI 0.28–0.66, p = < 0.00). Furthermore, quota and other refugee men had a lower likelihood of mental illness compared to their counterpart, however this association was not statistically significant (OR = 0.65 CI 0.34–1.23, p = > 0.05). Sex and legal status stratification showed that age was not associated with mental illness except for asylum seekers in the oldest age category, 36 and above (OR = 1.79 CI 1.09–2.94 p = < 0.0) and among men (OR = 1.64 CI 0.99–2.72, =<0.05). Stratifying by sex, the highest association for women was from lower-middle (OR = 4.01 CI 1.83–8.77, p = < 0.0) and upper-middle- and high-income countries (OR = 3.36 CI 1.76–6.40, p = < 0.01) but with large confidence intervals. Stratified analysis by legal status showed GNI classification (lower-middle and upper-middle-and high) to be significantly associated with mental illness among asylums seekers (OR = 2.87, CI 1.26-6-64, p = < 0.01; OR = 2.17, 1.03–4.53, p = < 0.05, respectively).

**Table 3:** Crude odds ratios (OR) for the associations between mental illness (score ≥ 11) sociodemographic characters, country of origin by World Bank GNI classification and largest groups by probability rate of being granted protection status in EU with 95% confidence intervals (CI), for total sample and stratified by sex and legal status

Crude odds ratios (OR) for mental illness					
	Total sample	Stratified by Sex		Stratified by Legal status	
	All	Men	Women	Asylum Seekers	Quota and other refugees
	**OR (95% CI)**	**OR (95% CI)**	**OR (95% CI)**	**OR (95% CI)**	**OR (95% CI)**
**Sex**					
Women	**1**			**1**	**1**
Men	0.52 (0.36–0.73) ***			0.43(0.28–0.66) ***	0.65 (0.34–1.23)
**Legal status**					
Asylum seekers	**1**	**1**	**1**		
Quota and other refugees	0.49 (0.34–0.71) ***	0.55 (0.33–0.89) **	0.36 (0.20–0.65) ***		
**Age**					
14–25	**1**	**1**	**1**	**1**	**1**
26–35	1.47 (0.96–2.24)	1.68 (0.98–2.86)	0.99 (0.48–2.03)	1.25 (0.75–2.08)	1.65 (0.73–3.73)
36>	1.73 (1.16–2.58) ***	1.64 (0.99–2.72) *	1.53 (0.77–3.05)	1.79 (1.09–2.94) *	1.37 (0.66–2.82)
**Country of origin by World bank GNI Classification**					
Low	**1**	**1**	**1**	**1**	**1**
Lower-middle	1.87 (1.23–2.84) *	1.42 (0.84–2.37)	4.01 (1.83–8.77) **	2.87 (1.26–6.64) **	1.01 (0.43–2.35)
Upper- middle and high	1.77 (1.19–2.61) *	1.16 (0.70–1.94)	3.36 (1.76–6.40) **	2.17 (1.03–4.53) *	1.54 (0.74–3.22)
**Probability rate of first-time recognition**					
High	**1**	**1**	**1**	**1**	**1**
Middle	2.21 (1.32–3.70) ***	1.82 (0.93–3.55)	2.82 (1.25–6.39) **	1.09 (0.62–1.91)	4.0 (1.15–13.91) *
Low	2.17 (1.41–3.32) ***	1.67 (0.97–2.87)	3.59 (1.77–7.31) ***	1.13 (0.66–1.94)	1.0 (0.17–5.64)

*p value < 0.05, **p-value < 0.01 ***p-value < 0.00

### Full model by legal status

#### Model 1

The full model (Goodness of fit test; χ^2^ = 9.047, df= (8), p = 0.33), (Table 4), when adjusting for age, sex and probability rate of first-time recognition showed that quota and other refugees had significantly lower odds of mental illness than asylum seekers (OR = 0.60 CI 0.38–0.93 p = 0.02). Coming from a country with middle and low probably of being granted asylum showed significant results (OR = 1.73 CI 0.99–3.03, p = 0.05 and OR = 1.64 CI 0.99–2.79, p = 0.05, respectively). Analyses showed all in the oldest age category were at significant higher risk of mental illness (OR = 1.55 CI 1.02–2.34, p = 0.03), and men were at lower risk (OR = 0.51 CI 0.36–0.73, p = 0.00) (not shown in table). Stratified by sex, when adjusting for variables in model 1 (Goodness of fit test; χ^2^ = 4.148, df= (7), p = 0.76), quota and other refugee women had significantly lower odds of mental illness than asylum seeking women (OR = 0.51 CI 0.26–0.99 p = 0.04). Also, women coming from a country with a low probability of being granted asylum showed had an increased risk of mental illness (OR = 2.67 CI 1.21–5.93 p = 0.01 (not shown in table). No other risk was evident in the model. Unlike for women, this model did not predict any differences among men (Goodness of fit test; χ^2^ = 6.941, df= (6), p = 0.32) (not shown in table).

**Table 4:** Adjusted odds ratios (OR) for mental illness (score ≥ 11) by legal status. Total sample and stratified by sex, presented with 95% confidence intervals (CI). Model 1 was adjusted for age, sex, and probability of getting asylum. Model 2 was adjusted for age, sex, probability of getting asylum, GNI classification as well as the interaction between sex and GNI classification

Adjusted odds ratios (OR) for mental illness by legal status						
	Model 1	**Model 2**	Model 1	**Model 2**	Model 1	**Model 2**
	All OR (95% CI) p-value	All OR (95% CI) p-value	Men OR (95% CI) p-value	Men OR (95% CI) p-value	Women OR (95% CI) p-value	Women OR (95% CI) p-value
**Legal Status**						
Asylum seeker	**1**	**1**	**1**	**1**	**1**	**1**
Quota and other refugees	0.60 (0.38–0.93) 0.02	0.60 (0.37-1.00) 0.05	0.65 (0.361.18) (NS)	0.52 (0.27–0.99) 0.04	0.51(0.26–0.99) 0.04	0.74 (0.33–1.6) (NS)

NS = Non Significant

#### Model 2

In model 2 (Goodness of fit test; χ^2^ = 6.510, df= (8), p = 0.59), when adjusting for GNI classification and interactions, quota and other refugees were still less likely to report mental illness (OR = 0.60 CI 0.38–0.93 p = 0.02). A significantly higher likelihood of mental illness was found among those with a middle probability of receiving a positive decision on the asylum application (OR = 1.89 CI 1.02–3.47, p = 0.04) and individuals in the oldest age category (OR = 1.68 CI 1.09–2.57, p = 0.01) (not shown in table). No difference in risk was evident in any of the variables.

In Model 2, stratified analysis by sex, (Goodness of fit test; χ^2^ = 8.273, df= (8), p = 0.40), we observed higher odds for asylum seeking men (OR = 0.52 CI 0.27–0.99 p = 0.04). Additionally, men in the oldest age category were at higher risk of reporting mental illness (OR = 1.75 CI 1.02–2.98 p = 0.04) (not shown in table). For men, no other risk was evident in the model. When adjusting for variables in model 2, no association was observed for women (Goodness of fit test; χ^2^ = 4.974, df= (8), p = 0.07).

## Discussion

The aim of this study was to estimate the differences in prevalence of mental illness between asylum seekers and refugees, including both quota and other refugees. We found quota and other refugees to be at a 40% lower risk of mental illness compared to asylum seekers, confirming our hypothesis. We also assessed whether sex, age, gross national income per capita in the country of origin, or by probability of being granted asylum could explain the differences in mental health among the groups. Our hypothesis was not fully confirmed. Although these variables partly influenced the prevalence of mental illness, they did not explain the prevalence in a consistent way.

Our results depict that asylum seekers have a specifically high likelihood of mental illness compared to other refugees such as quota and those coming under other prerequisites. Previous studies have shown that asylum seekers are at increased risk of developing mental illnesses, but these studies have often compared asylum seekers with migrants who have been in the country for a longer period, and as a result, potentially made the comparison possibly biased [2, 26, 27]. The increased risk for asylum seekers found in our study could be partly explained by their uncertain residence status and uncertainty regarding their asylum application [28–31]. Asylum seekers are confronted with more unfavorable circumstances when it comes to, for instance, housing opportunities, health care education and working conditions, compared to those who have been granted residence permit. Legal status has shown to be a significant predictor of poor mental health, even when controlling for pre-and post-migration factors such as traumatic events, post-migration resources and social desirability [26, 32]. Remarkably, severe levels of mental illness were more commonly reported than mild and moderate levels of mental illness, for both groups, 52.9% and 35.7% respectively, indicating higher intensity of symptoms.

Our finding also corroborates previous literature on the differences in risk of mental illness by country or region of origin. Regional differences in reported mental illness, experienced traumatic events and use of psychiatric care have been found [32–35]. In addition, we found differences in prevalence and likelihood of mental illness by GNI classification. For instance, individuals from Iraq and Iran (upper- middle- and high-income countries) were at elevated risk compared to individuals from Afghanistan and Somalia (low-income countries). Furthermore, those originating from Cameron and Senegal (lower-middle income countries) were at higher risk compared to those originating from Syria (low-income country).

A recent Finish study found that asylum seekers from Sub-Saharan Africa reported more traumatic events than asylum seekers from other regions [32]. Moreover, refugees from Syria, Iraq, and Afghanistan were most likely to screening positive for mental health symptoms compared to refugees from other countries such as Somalia, Myanmar and D.R. Congo [33]. We did not find elevated likelihood for Syrian asylum seekers, quota, and other refugees, which accounted for the largest groups in our sample. However, during the time of the study, Syria was classified as a low-income country according to the World Bank and not as a lower-middle income country as previously done. Using the previous classification might have yielded different results.

We have used country of origin by GNI classification, a global classification to portray social and economic differences and similarities across the sample. Presenting country of origin based on GNI might give a more representable picture, as countries based on region classification for example (MENA region, Asia, Europe) impose problems with heterogeneity, as there is within region differences in terms of social development and access to health care. Our study provides a way to classify countries and identify trends in other areas than only country of origin.

Nevertheless, differences in mental illness have been postulated to be explained by other factors than region of origin such as socioeconomic factors, cultural differences, differences in social support and coping style, discrimination, and varying vulnerabilities among different subgroups [7, 14, 33].

Our study expands on prior research examining differences by sex and age, with women reporting higher odds for mental illness, compared to men. Both female sex and older age have been linked with poorer psychological health in refugees, though there are some studies that demonstrate no effect of sex on mental illness [14, 33, 36–38]. It has been suggested that the sex differences in mental health are linked to roles and social positions, where women are limited in their role and lack of choice. Literature has been devoted to explaining the sex gap because of exposure to trauma and lack of self-control. Indeed, the broader social determinants of health associated with restricted economic opportunities, insecure housing, location of residence and migration status can have a profound influence on one’s sense of control [14, 38, 39]. It could also be that women may be at higher risk due to increased sexual victimization and domestic violence. Considering age, our results are consistent with past work, inciting greater vulnerability in older people. This could be due to greater accumulation of traumas over time [7, 33] for older people, or younger refugees being less affected by the enduring stresses of displacement.

Determination of illness severity could have important clinical implications when it comes to, for example treatment strategies or prioritizing care when resources are scares. Considering this aspect may be useful in planning public health interventions targeting this vulnerable group. Furthermore, our findings could be applicable to individuals without legal document/status (former asylum seekers who have been rejected asylum/or others without legal status) as these individuals are often confronted with similar challenges and have same health care entitlements as asylum seekers.

## Limitations and strengths

The study has many strengths: first, data is generated through a validated screening instrument specifically developed for refugee populations. Although the study was conducted only in one region in Sweden, it was conducted in two primary healthcare centers that together conducts a high number of HE in Stockholm, in addition to including both asylum seekers and other refugees, which increases the generalizability of the findings. Both asylum seekers and other refugees are represented in our study, giving the study a good representation in terms of migrants. Data was collected with the help of health care professionals working in the centers and there was no need for outreach methods, which minimizes the risk of bias or misunderstanding of the data.

While this study provides important information on differences in mental illness by legal status there are several limitations. We had no information on risk and resilience factors related to participants’ mental health such as trauma experiences, length of stay in Sweden, educational level and other known determinants of mental health. Another limitation is possible selection bias as the RHS-13 screening was part of a voluntary HE and those opting out of HE might have different mental health profiles. In addition, we do not have information about the few who took part in an HE but declined screening with the RHS-13. It could be that their mental health profiles too were different from those that accepted screening. Furthermore, the demographics of those coming to Sweden during this period might not be stable and generalizable to other time periods in the future as migration patterns can change, for instance, in terms of reasons for migrating, country of origin and sociodemographic factors. Unlike Sweden, other countries might offer different entitlements in terms of housing and health care access to asylum seekers and other refugees which might have impact on their mental health.

Additionally, the rational for using screening as opposite to diagnostic testing in our study was to identify disease in an early and pre-symptomatic stage, rather than using a diagnostic test to show symptoms of a specific diagnosis, such as PTSD, depression, or other mental illness conditions.

This is mainly due to the complexity of the population under study. Choosing to only concentrate on a certain disease, for example PTSD, would have increased the risk of missing other mental illness conditions, at the same time leading to ethical issues in balancing the benefits and harms of testing an already vulnerable group, as we would then assume all asylum seekers and other refugees coming for a HE suffer from one condition (for example, PTSD).

Furthermore, though many of the diagnostic measures have been widely used in different cultural contexts, none have been specifically developed for refugee populations or cross-cultural use [9, 11]. While diagnostic interviews are the gold standard for validations, they were not feasible in this study given the limited resources within the HE and we aimed for a low threshold strategy that was relatively easy to implement in the existing structure.

However, it could have been valuable to complement the screening with RHS-13 with a diagnostic instrument or other proxy once the patient scored positive for mental illness. Nevertheless, one limitation of using the RHS-13, as with other screening programs might be that estimation of benefit from early screening may be influenced by length-time bias. We had no data on length of stay in Sweden. It could be the case that the timing of screening during the HE is not optimized in terms of case finding, as mental illness can change over time. Preferably a follow-up screening, 6 months after an initial HE would have been valuable.

## Conclusion

Legal status moderated mental illness. Asylum seekers were at greater risk of mental illness compared to quota and other refugees. Legal status as well as female sex and region of origin were associated with migrant’s mental health. Additionally, consideration of legal status should be considered to better understand and plan interventions targeting asylum seekers and refugees’ mental health.

In order to prevent mental illness among asylum seekers and other refugees, effective public mental health responses may be put in place. Presence of mental illness among asylum seekers and other refugees can negatively impact the integration and adaptation in the host country. Integrating low threshold mental health services facilitate early detection and identification of mental illness, while taking into consideration the heterogeneity of refugees in terms of sociodemographic factors and legal status of the person. Further research is needed to better understand and address the diversity of mental illness by legal status.

## Data Availability

The data that supports the findings of this study are available from the two participating health care clinics in region Stockholm, but restrictions apply to the availability of these data, which were under license for the current study, and so are not publicly available. Data are however available from the authors upon reasonable request and with permission of the Swedish Ethical Review Authority and Region Stockholm. For data request contact fouu.slso@regionstockholm.se.

## Abbreviations

EU: European Union
GNI: Gross National Income
HE: Health examination
MR: Medical record
PTSD: Post-traumatic stress disorder
RHS-13: Refugee Health Screener
UN: United Nations
UNHCR: United Nations High Commissioner for Refugees
